# Computational analysis of the relationship between allergenicity and digestibility of allergenic proteins in simulated gastric fluid

**DOI:** 10.1186/1471-2105-8-375

**Published:** 2007-10-09

**Authors:** Bingjun Jiang, Hong Qu, Yuanlei Hu, Ting Ni, Zhongping Lin

**Affiliations:** 1 College of Life Sciences, National Lab of Protein Engineering and Genetic Engineering of Plants, Peking University, Beijing 100871, P. R. China

## Abstract

**Background:**

Safety assessment of genetically modified (GM) food, with regard to allergenic potential of transgene-encoded xenoproteins, typically involves several different methods, evaluation by digestibility being one thereof. However, there are still debates about whether the allergenicity of food allergens is related to their resistance to digestion by the gastric fluid. The disagreements may in part stem from classification of allergens only by their sources, which we believe is inadequate, and the difficulties in achieving identical experimental conditions for studying digestion by simulated gastric fluid (SGF) so that results can be compared. Here, we reclassify allergenic food allergens into alimentary canal-sensitized (ACS) and non-alimentary canal-sensitized (NACS) allergens and use a computational model that simulates gastric fluid digestion to analyze the digestibilities of these two types.

**Results:**

The model presented in this paper is as effective as SGF digestion experiments, but more stable and reproducible. On the basis of this model, food allergens are satisfactorily classified as ACS and NACS types by their pathways for sensitization; the former are relatively resistant to gastric fluid digestion while the later are relatively labile.

**Conclusion:**

The results suggest that it is better to classify allergens into ACS and NACS types when understanding the relationship between their digestibility and allergenicity and the digestibility of a target foreign protein is a parameter for evaluating its allergenicity during safety assessments of GM food.

## Background

IgE-mediated allergy is an immunoreaction that occurs when the immune system reacts improperly to otherwise innocuous proteins, designated allergens. Binding of an allergen to IgE attached to mast cells and basophils, stimulates the release of inflammatory substances, such as histamine, leading to disease reactions. Such reactions can involve a variety of symptoms, such as pins and needles, swelling of the oral cavity, alimentary canal and respiratory tract reactions or anaphylaxis. According to the American Academy of Allergy, Asthma and Immunology (AAAAI), there are about 40–50 million allergy sufferers in the USA, making it the sixth most common cause of chronic illness [1], and food allergies occur in 6%–8% of children under 4 years old [1] and in 4% of adults [2]. Therefore, allergy has become a major clinical and public health issue.

Allergens occur in many foods, such as rice, hen's egg and peanut as well as in a number of non-food sources, typified by fungi, mites and insects. The Food and Agriculture Organization (FAO) and the World Health Organization (WHO) proposed that milk, seashell, egg, fish, peanut, soybean, nut and wheat are eight major sources of food allergens and the causes of most food allergies [3]. These allergens have attracted much attention, and efforts have been made to determine the reasons for their allergenicity and what we can do to reduce it or to remove them. The arrival of genetically modified organisms (GMO) has made it even more urgent to find an accurate method for evaluating allergenicity as a part of the safety assessment of GMO before they are released commercially. Astwood et al. found that the main allergens of peanut and soybean were more stable in simulated gastric fluid (SGF) than nonallergenic proteins such as spinach ribulose bis-phosphate carboxylase/oxygenase, and proposed that the resistance of a protein to digestion is an important factor in its allergenicity [4]. After that, assessment of the digestibility of a foreign protein in GM foods as a means of evaluating allergenicity was included not only in a decision tree approach proposed by FAO and WHO in January 2001 [3], but also in a weight-of-evidence approach proposed by The Codex Alimentarius Commission (Codex) in 2003 [5]. Several ensuing studies have shown that allergenic proteins are more stable to pepsin digestion than those not connected with allergenic potential, but Yagami et al. [6] and Fu et al. [7,8] found that the association between digestibility and allergenicity is not absolute. The disagreements may be attributed to variations in the amount of enzyme or protein, pH, temperature, ionic conditions etc. in the experiments. Computational simulation has the potential to substitute for manual experiments and will always achieve the same (simulated) conditions. However, there has been no report on computational simulation of gastric fluid digestion. Therefore, we have considered computer simulation of gastric fluid digestion to elucidate the relationship between the allergenicity and digestibility of proteins.

To date, about 256 food allergens have been included in the SDAP, including 121 allergens with known amino acid sequences and 10 with known allergenic determinants [9,10]. The food and pollen allergen database in our laboratory also includes the amino acid sequences of major food and pollen allergens from eight species [11]. These data make it possible to simulate gastric fluid digestion by a computer model. Food allergens can enter the human body either through ingestion or – when also occurring as airborne substances – by inhalation. Accordingly, these allergens can be divided into two groups as defined by the pathway of entrance leading to sensitization. Non-food allergens, however, mainly enter by the airborne pathway. Ingested allergens can be dubbed alimentary canal-sensitized (ACS), while others can be dubbed non-alimentary canal-sensitized (NACS). We propose that these two allergen groups would respond differently to gastric fluid digestion: the former is likely to be relatively resistant and the latter relatively labile. However, allergens are generally ingested along with nonallergenic proteins, so they must differ somewhat in biochemical properties. Proteins from SwissProt were collected into their respective species-origin protein sets (SOPS), and the digestibilities of allergens and SOPS were compared in an effort to derive an in silico model, which attempts to simulate protein susceptibility to gastric/intestinal protease activity.

## Results

We compared the results from digestion of food allergens and of relevant SOPS to estimate the resistance of allergens to gastric fluid digestion. Then we compared the results from digestion of ACS and NACS food allergens to establish a provisional threshold for assessing the digestibility of target proteins. Finally, we used non-food allergens from SDAP to test this model.

### Comparison between the digestibility of food allergens and relevant SOPS

**Figure 1:**
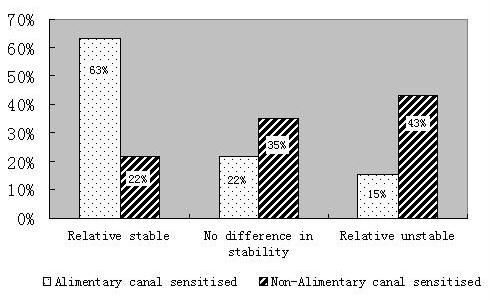
**The ratio taken up by food allergens with different stabilities in the digestion model**. ACS food allergens labelled by the spotted bar are more stable than NACS food allergens, labelled by the biased bar.

Results from the in silico digestion study, involving food allergens and SOPS (listed in the additional files 1 and 2), were compared using the t test (p = 0.01). The percentages of ACS or NACS food allergens that were digested more or less efficiently than the relevant SOPS are shown in Figure 1. We found about 63% of ACS food allergens are relatively resistant and only 15% are relatively labile, whereas the corresponding figures for NACS food allergens are 22% and 43%, respectively (Figure 1). The difference is significant; it indicates that ACS food allergens are relatively resistant and NACS food allergens relatively labile to digestion by gastric fluid. It also suggests that allergens cannot be divided into food and non-food types solely on the basis of their sources. Classification into ACS and NACS allergens on the basis of the pathway of sensitization seems more satisfactory. Therefore, in future evaluations of genetically modified food, a better approach will be to perform assessments according to the pathway through which the foreign proteins enter the human body. Such an approach is expected to produce more reliable results.

### Comparison between the digestion of ACS and NACS food allergens

**Figure 2:**
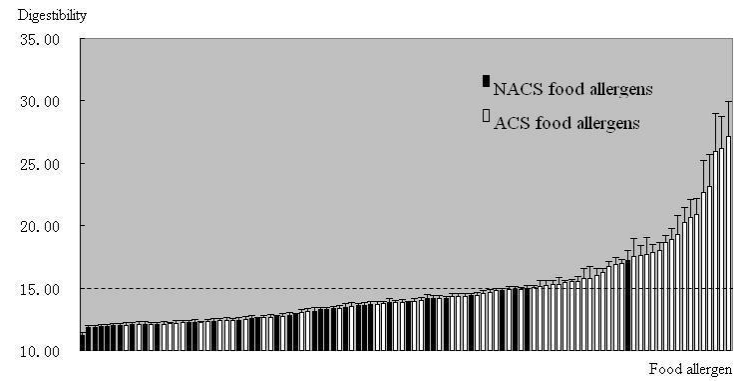
**Digestibility of food allergens**. ACS food allergens are labelled by the white bar and NACS by the black bar. The dashed line shows the threshold of digestibility at 15.00. The unit for digestion results is the amino acid residue.

Figure 2 shows the results in the additional files 1 and 2 arranged by the lengths of the digestion products. A threshold can be set at 15.00 amino acid residues. Although this is somewhat arbitrary, we found that most of the digestion results are below the threshold for NACS food allergens, while for ACS food allergens, about a half the digestion results are above it. Moreover, most of the allergens with results above the threshold are ACS, while only one NACS allergen is above the threshold. This suggests that we can use the threshold to assess the digestibility of a target protein using the model.

### SDAP non-food allergens are relatively labile to digestion

**Figure 3:**
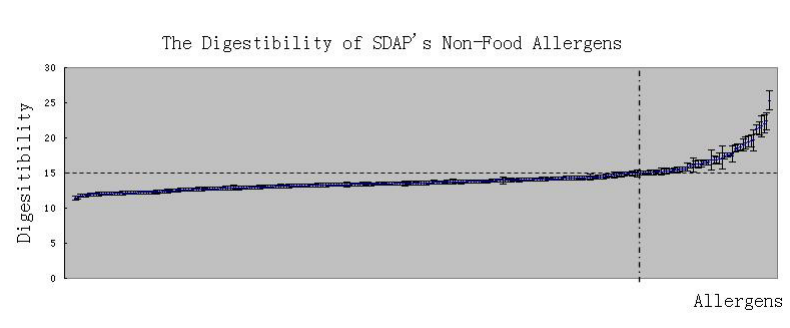
**Digestibility of SDAP non-food allergens**. The horizontal dashed line shows the threshold of digestibility at 15.00, and the vertical dashed line shows the border, to the left of which the digestibility is lower than the threshold while to the right it is higher. The unit for digestibility is the amino acid residue.

SDAP non-food allergens should be NACS according to our classification so they should be relatively labile to digestion by gastric fluid. We used the model to assess the digestibility of SDAP non-food allergens; the results are listed in the additional file 3. These results are sorted and shown in Figure 3: 215 allergens are below the threshold of 15, while only 49 are above. This suggests that SDAP non-food allergens are relatively labile, consistent with our expectation.

## Discussion

### The digestion model

**Figure 4:**
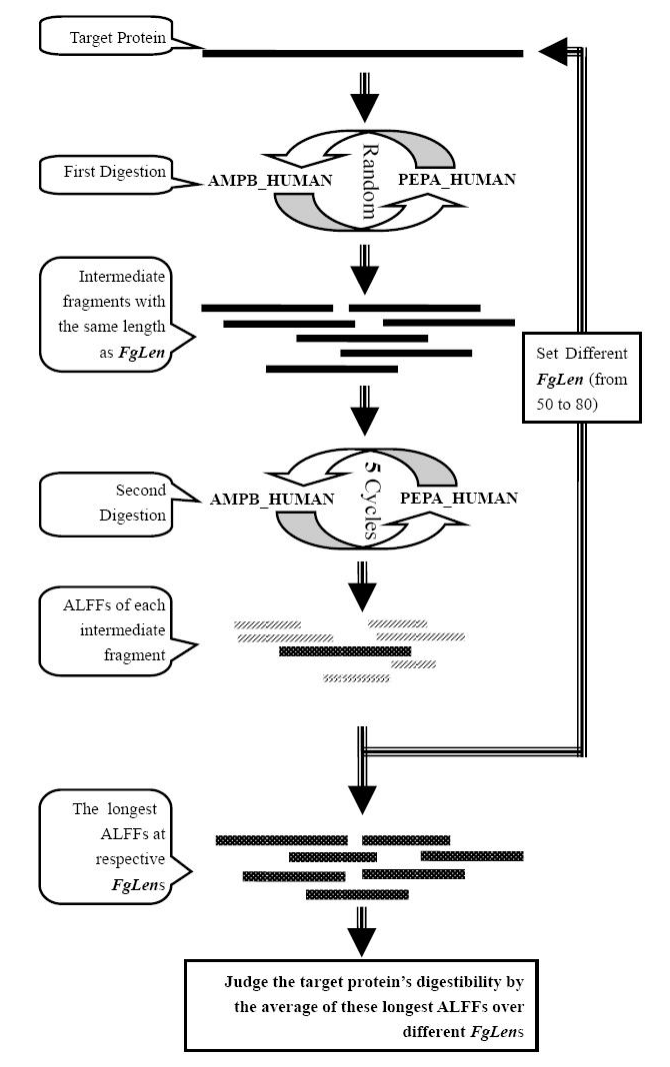
**Digestion Model**. The model has two digestion steps. In the first, the target protein is digested into intermediate fragments with the same length as FgLen. In the second, these intermediate fragments are further digested and the respective ALFFs are calculated. The longest ALFF is selected, shown as the dotted line. FgLen is changed from 50 to 80, the longest ALFF at different FgLen is collected into a set, and the average of the set is used to evaluate the resistance of the protein to gastric fluid digestion.

In the development of this model, we found that sequence length had a significant impact on the final fragment length when a one-step digestion was used. Therefore we tried to segment the protein sequences into fragments with the same lengths for further digestion in order to eliminate the sequence length effect. Initially, we tried using a sliding window to obtain overlapping fragments, but this proved too time-consuming. The two-step digestion model was then designed as shown in Figure 4; this yields results comparable with the former method but much more quickly. In the first digestion, the target protein is cut into many intermediate fragments with the same length as FgLen, one parameter in the model. The average length of the final fragments (ALFF) can then be calculated for each intermediate fragment after the second digestion. One target protein can yield as many ALFFs as intermediate fragments. However, any one protein can be divided into regions with different digestibilities, some regions more sensitive to digestion and others more resistant. We believe that simulated digestibility of the most resistant region can be used to determine in silico digestibility of the whole protein. Therefore, the largest ALFF is selected for a given FgLen, and the average of these largest ALFFs over different FgLens should measure the digestibility of the target protein.

The model has two parameters, FgLen for the first digestion and iterative cycles for the second. FgLen is the length of the intermediate fragments produced by the first digestion, which is set in the range 50–80. From SDAP, the epitopes of allergens are generally 8–25 amino acids in length and would be contained in the final fragments after digestion. For many proteins, we found that if FgLen was set at less than 50, the ALFF were less than 8 amino acids. Therefore, the lowest FgLen value was set at 50. The highest value was set at 80 in order to include as many allergens as possible, because some allergens are about 80 amino acids long. As for the iterative cycles, an arbitrary set of proteins was tested to produce a curve of the outcomes of the iteration cycles of the second digestion, and we found that after 7–13 iteration cycles the outcomes approached a constant limit. Therefore, the setting for the iteration steps of the second digestion was 5. Using some allergens and relevant SOPS, we also tested numbers of iterative cycles from 3 to 7, and obtained similar conclusions about digestibility; that is, the finding that protein A is more stable than protein B was always consistent irrespective of the number of iterative cycles from 3 to 7. This indicates that the setting of iterative cycles has no significant effect on the final results, which could be attributed to the strategy for assessing the digestibility of the target protein.

Allowing for possible differences in pepsin activity in vivo, the model used not only PEPA_HUMAN, pepsin in gastric fluid, but also AMPB_HUMAN, an aminopeptidase that was introduced to enhance the model so that it simulates the complex gastric fluid environment. There are usually fewer sites in a protein that are susceptible to AMPB_HUMAN than to PEPA_HUMAN; therefore, the introduction of AMPB_HUMAN will not cause large errors. And as we know, the structure of a protein affects its digestibility by gastric fluid. Sen et al. found that Ara h 2 has a compact structure that make it resistant to pepsin digestion [12]. However, there is less information about the relationship between compact structure and digestibility. At present, protein 3D-structure cannot be predicted reliably, so it is very difficult to introduce protein 3D-struture into the model. However, most proteins will be denatured in a low pH environment such as gastric fluid, and during the initial sensitization to an allergen, the fragmentation of a protein into short fragments is required to induce antibody production. Therefore, the model treats all proteins as linear units and all enzyme target sites are equally accessible without leading to large errors. For example, the final digestion result for Ara h 2 is 20.27 ± 1.21, which is in the top ten of all allergens tested and suggests resistance to gastric fluid, consistent with Sen et al. [12]. It can get the result that the main allergens of peanut and soybean are resistant to SGF digestion while spinach ribulose bis-phosphate carboxylase/oxygenase is labile, which is consistent with Astwood et al. [4]. It also indicates that Bet v 1 is relatively labile and Ara h 6 is relative resistant, as shown experimentally by Breiteneder [13] and Suhr et al. [14]. These suggest that the model could yield results comparable to those from manually simulated gastric fluid digestion experiments. The result – that ACS food allergens are more resistant than NACS food allergens – is consistent with our expectation. SDAP non-food allergens should be NACS according to the classification by sensitized pathway and the result – that they are labile to SGF digestion – is also consistent with our expectation too. These further corroborate the reliability of the model. When assessing the allergenicity of bacterial codA, Singh et al. found it could be completely degraded with SGF [15], and the model can also get a similar result with the average 14.47 ± 0.21 less than the threshold. And for the amaranth 11S globulin, the model can also get a similar result as Sinagawa-Garcia SR et al. got when assessing the safety of genetically modified maize [16]. These also corroborate the reliability of the model and suggest that the model could be used at the safety assessment of GM food [17].

### The classification of allergens

Allergens are usually classified into food and non-food types. This classification ignores differences in the pathway of sensitization to allergens and leads to difficulties in evaluating the relationship between the digestibility and allergenicity of allergens. According to the results of this study, classification by the pathway of sensitization is more reliable and reasonable. ACS allergens usually enter the human body with food through ingestion; it is to be expected that they are more resistant to digestion by the gastric fluid. However, NACS allergens often enter the body via an airborne pathway and therefore are less resistant to digestion. This is supported by the present results. Therefore, the resistance of ACS allergens to gastric fluid digestion influences their allergenicity to some extent. However, NACS allergens can trigger allergy before digestion by gastric fluid. They are even more labile to digestion by gastric fluid. This is consistent with the conclusion of Breiteneder and Clare Mills that the stabilities of allergens from the plant prolamin and cupin superfamilies are relatively high while that of the plant pollen Bet v 1 family is relatively low [13]. Results derived from our in silico digestion model clearly indicate the ACS allergens as being relatively resistant to proteolytic degradation, whereas those of the NACS category do not appear markedly stable. The results suggest that, when the allergenicity of GM food is evaluated, the ACS and NACS pathways for target proteins should be distinguished to avoid confusion in experimental simulations of digestion. For example, Vieths et al. found that peanut allergens are relatively resistant while hazelnut allergens are relatively labile [18]. One possible explanation is that the peanut allergens belong to the ACS allergen group while the hazelnut allergens are NACS according to our classification.

In the additional file 1 shows examples of ACS food allergens that are relatively labile, perhaps for the following reasons. First, insufficient data are available to allow the pathways of sensitization to allergens to be distinguished reliably. We classified food allergens as NACS according to the literature, but few publications could help us to identify ACS food allergens. We had to assume that food allergens that could not be classified as NACS on the basis of available evidence should be classed as ACS. Inevitably, some food allergens will be incorrectly classified as ACS. Second, in designing the digestion model, variations in age and region were not considered. For example, those who are sensitive to the α-lactalbumin protein Bos d 4 and β-lactoglobulin Bos d 5 in milk are mostly children. Adults are rarely sensitive to either of these proteins. The reason may be that the ability to digest them is lower in children than in adults. The two proteins seemed easier to digest according to the model if age difference was not considered. Non-specific lipid transfer proteins such as Mal d 3, Lyc e 3 and Zea m 14 are easy to digest according to the model, but some reports suggest that they are fairly resistant to pepsin [19]. This may be related to the region in which allergies occur: they generally result in allergy in the Mediterranean population [20], but the digestion model does not account for regional differences. Apart from the above examples, carp recombinant allergen rCyp c 1.01, which is easy to digest according to the model, has a similar character to respiratory tract allergens [21]. This protein may be related to sensitization via the respiratory tract.

By comparing the digestibilities of ACS and NACS food allergens, we can set the threshold of digestibility at 15.00. We used SDAP non-food allergens to test the model and the threshold and found that most non-food allergens were relatively labile, which is consistent with our expectation. We also found that some allergens were very stable, such as Ani s 2 and Ani s 3, which are from the fish parasite Anisakis simplex [22]. Although people usually do not intend to eat the parasites, they may be consumed along with fish, and Ani s 2 and Ani s 3 are ACS allergens to some extent. Therefore, the model can be reliably used to assess the digestibility of target proteins.

All the results suggest that the stabilities of allergens differ among different allergenic patterns and pathways. This is an important criterion for the analysis of allergenicity in the assessment of safety of genetically modified food. In other words, we should consider the allergenic pathway, i.e. non-alimentary canal-sensitized (airborne pathway) versus alimentary canal-sensitized. In addition, the stabilities of SOPS are different, indicating that when the resistance to digestion or allergenicity of any protein is analyzed, the environmental condition should be taken into account to avoid possible errors. Finally, the model can quantify the digestibility of a target protein; therefore it can be combined with other methods to produce a complex model for predicting the allergenicity of a target protein, to change the digestibility of a target protein by a mutation that does not alter its activity, or to assess the safety of GM food.

## Conclusion

Results presented in this paper show that our simulated (in silico) digestion model, based on two distinct gastric enzymes, can – on a general level – reveal dissimilarities between food allergens of the alimentary canal-sensitizing (ACS) type and non-allergens. A bifurcation of food allergens into ACS and non-alimentary canal sensitizing (NACS) proteins was required to clearly demonstrate this pattern, since the latter group did not disclose appreciable protease resistance in the aforementioned in silico digestion model. Hence, the former class only may be adequate for this sort of assessment. Further investigations are, however, needed to show if these findings are generally supported by in vitro simulated gastric fluid assay.

## Methods

### Collecting and pre-processing of protein data

Allergen data were obtained from SDAP. Food allergens were classified into two groups according their pathway for sensitization. They were classified as NACS according to the literature (Table 1), but few publications could help to identify ACS food allergens. Therefore, all food allergens that could not be classified as NACS on the basis of the literature were considered ACS. Non-food allergens that should be NACS according to the classification by the pathway of sensitization were also collected from SDAP. However, allergen sequences shorter than 80-amino acid residues were discarded and cd-hit [23] was used to delete redundant sequences for each allergen.

**Table 1:** NACS food allergens

Food allergen	Reasons to list as NACS allergens
Act c 1	Airborne*
Api g 1	The celery homologue of the major birch pollen allergen, Bet v 1 [25, 26]
Api g 4	High homology to pollen allergen, Bet v 2 [27, 28]
Api g 5	Pollen-related [29]
Ara h 5	High homology to pollen allergen, Hev b 8.0102
Ara h 8	Homology to pollen allergen Bet v 1 [30]
Bos d 2	Non-food allergen*
Bos d 3	Non-food allergen*
Bra n 1	Aeroallergenic protein [31]
Bra n 2	Calcium-binding pollen allergen
Bra r 1	Calcium-binding pollen allergen
Cap a 2	Pollen related [32]
Car p 1	Airborne*
Cor a 1	Hazel pollen allergen
Cor a 2	Homologous with the birch pollen allergen Bet v 2*
Dau c 1	Homologous with the birch pollen allergen Bet v 1*
Dau c 4	Homologous with the birch pollen allergen Bet v 2*
Gly m 1	Airborne, soybean-hull dust
Gly m 2	Soybean hull allergen
Gly m 3	Homologous with the birch pollen allergen Bet v 2*
Gly m 4	Homologous with the birch pollen allergen Bet v 1*
Hor v 1	Flour allergen causing baker's asthma disease
Hor v 9	Pollen allergen^§^
Lit c 1	Homologous with the birch pollen allergen Bet v 2*
Lyc e 1	Homologous with the birch pollen allergen Bet v 2*
Lyc e LAT52	Anther specific LAT52 protein*
Mal d 1	Homologous with the birch pollen allergen Bet v 1*
Mal d 4	Homologous with the birch pollen allergen Bet v 2*
Mus xp 1	Homologous with the birch pollen allergen Bet v 2*
Ory s 1	Pollen allergen*
Ory s 33 kD	Airborne [33]
Sola t 1	Pollen related [34]
Tri a 3	Pollen allergen-like^§^
Tri a profilin	Homologous with the birch pollen allergen Bet v 2*
Tri a ps93	pollen allergen homolog^§^
Tri a TAI	Major allergen of wheat flour responsible for baker's asthma^§^
Zea m 1	Expressed in anthers and pollen^§^

^§^: GenBank. : SwissProt.*: SDAP.

Protein sequences were selected from SwissProt according to the species origin of food allergens, and made up the respective SOPS. Protein sequences shorter than 80 or longer than 500 amino acid residues were discarded from each SOPS. Redundant sequences were eliminated from each SOPS by cd-hit [23]. The SOPS of some species such as cattle and rice were too large, and protein sequences were arbitrarily deleted from them until about 2000 remained.

### Establishment of computational model

Two proteolytic enzymes, PEPA-HUMAN and AMPB-HUMAN, were selected from the ExPASy ENZYME database [24] by their properties (Table 2). PEPA-HUMAN is common pepsin in human gastric fluid and specifically hydrolyzes peptide bonds of hydrophobic residues, especially aromatic ones. AMPB-HUMAN is a gastric amino-peptidase that specifically hydrolyzes basic amino-terminal residues. It was included to increase the randomness of the model and to reflect the complexity of human gastric fluid digestion.

**Table 2:** Human enzymes used in the model

Enzyme	Classification	Activity
PEPA_HUMAN	3.4.23.1	Specifically hydrolyzes peptide band involving a hydrophobic amino acid residue, especially an aromatic amino acid residue
AMPB_HUMAN	3.4.11.6	Release of N-terminal Arg and Lys from oligopeptides when P1' is not Pro

As shown in Figure 4, the model has two digestion steps. At one digestion cycle, the model will arbitrarily select one enzyme for one protein or each one of the fragments produced in the previous cycle, and will arbitrarily select one of the possible active sites of the selected enzyme and digest the substrate into two fragments. In the first step, the parameter FgLen is set to control the length of intermediate fragments produced by the digestion cycle. Intermediate fragments shorter than FgLen are discarded, while those longer than FgLen are redigested until their length is FgLen. Therefore, many intermediate fragments with the same length of FgLen will be produced in the first digestion step. During the second step, the model digests each intermediate fragment produced in the first step for only five cycles, regardless of the length of the final fragments. Final fragments with different lengths will be produced for each intermediate fragment after the second digestion and the ALFF can be calculated. From the ALFFs of the intermediate fragments, the longest is selected to represent the digestibility of the target protein for a given FgLen. By repeating the digestion steps with FgLen varying from 50 to 80, the average of the longest ALFFs at different FgLens provides a measure of the resistance of the protein to gastric fluid digestion. Large averages indicate relatively stable proteins and vice versa.

## Authors' contributions

BJ was the main investigator and writer. HQ wrote part of the method section. ZL contributed ideas. YH was in charge of National Basic Research Programming 973 for the assessment of GMO allergenicity. NT provided the allergen database. All authors read and approved the final manuscript.

## Supplementary Material

Additional file 1 The digestibility of ACS food allergens and relevant SOPS. The data provided represent the digestibility of ACS food allergens and relevant SOPS. Click here for file

Additional file 2 The digestibility of NACS food allergens and relevant SOPS. The data provided represent the digestibility of NACS food allergens and relevant SOPS. Click here for file

Additional file 3 Digestibility of SDAP non-food allergen. The table lists the digestibility of SDAP non-food allergen evaluated by the model. Click here for file
