# Supplementary material for: Computational analysis of the relationship between allergenicity and digestibility of allergenic proteins in simulated gastric fluid
Source: BMC Bioinformatics. 2007 Oct 9;8:375. doi: 10.1186/1471-2105-8-375 (PMC2099448; doi:10.1186/1471-2105-8-375)
Supplement: Additional file 1 — The digestibility of ACS food allergens and relevant SOPS. The data provided represent the digestibility of ACS food allergens and relevant SOPS. [file 1471-2105-8-375-S1.doc]

## Table 3 - The digestibility of ACS food allergens and relevant SOPS

Digestibility: the unit is the amino acid residue.

Comparison: comparing the digestibility of ACS food allergens with the digestibility of relevant SOPS by t-test (α= 0.01).

| Allergens | Digestibility | Species | Digestibility | Comparison |
| --- | --- | --- | --- | --- |
| Ara h 1 | 20.89±1.29 | *Arachis hypogaea* | 13.61±0.04 | Greater |
| Ara h 2 | 20.27±1.21 |  |  | Greater |
| Ara h 3 | 20.62±1.51 |  |  | Greater |
| Ara h 4 | 19.30±1.55 |  |  | Greater |
| Ara h 6 | 15.30±0.28 |  |  | Greater |
| Ara h 7 | 17.67±1.36 |  |  | Greater |
| Bos d 4 | 13.40±0.20 | *Bos domesticus* | 13.98±0.01 | Smaller |
| Bos d 5 | 13.04±0.23 |  |  | Smaller |
| Bos d 6 | 15.05±0.19 |  |  | Greater |
| Bos d 8 | 14.77±0.18 |  |  | Greater |
| Bra j 1 | 16.25±0.31 | *Brassica juncea* | 13.46±0.03 | Greater |
| Bra r 2 | 14.59±0.18 | *Brassica rapa* | 13.62±0.03 | Greater |
| Cha f 1 | 18.91±0.86 | *Charybdis feriatus* | 13.81±0.33 | Greater |
| Cor a 10 | 16.93±0.52 | *Corylus avellana* | 13.94±0.14 | Greater |
| Cor a 11 | 17.53±1.48 |  |  | No diff |
| Cor a 8 | 12.72±0.26 |  |  | Smaller |
| Cor a 9 | 16.74±0.43 |  |  | Greater |
| Cra g 1 | 25.97±3.03 | *Crassostrea gigas* | 13.62±0.05 | Greater |
| Cyp c 1 | 12.41±0.15 | *Cyprinus carpio* | 13.82±0.03 | Smaller |
| Fag e 1 | 17.88±0.59 | *Fagopyrum esculentum* | 13.95±0.11 | Greater |
| Fag e 8kD | 14.85±0.24 |  |  | Greater |
| Gad m 1 | 12.30±0.16 | *Gadus morhua* | 11.68±0.07 | Greater |
| Gal d 1 | 14.31±0.22 | *Gallus gallus* | 12.53±0.11 | Greater |
| Gal d 2 | 13.88±0.12 |  |  | Greater |
| Gal d 3 | 14.88±0.18 |  |  | Greater |
| Gal d 4 | 13.73±0.16 |  |  | Greater |
| Gal d 5 | 14.44±0.19 |  |  | Greater |
| Gal d apovitellenin | 12.07±0.22 |  |  | No diff |
| Gal d vitellogenin | 12.09±0.18 |  |  | No diff |
| Hal d 1 | 26.16±2.55 | *Haliotis diversicolor* | 14.55±0.52 | Greater |
| Hel as 1 | 23.12±2.57 | *Helix aspersa* | 13.76±0.30 | Greater |
| Hom a 1 | 18.64±0.59 | *Homarus americanus* | 13.26±0.11 | Greater |
| Hor v 21 | 14.35±0.25 | *Hordeum vulgare* | 13.42±0.01 | Greater |
| Gly m Bd28K | 14.29±0.31 | *Glycine max* | 13.69±0.01 | No diff |
| Gly m conglycinin | 16.97±0.35 |  |  | Greater |
| Gly m glycinin G1 | 17.60±0.81 |  |  | Greater |
| Gly m glycinin G2 | 16.01±0.55 |  |  | Greater |
| Gly m lectin | 13.92±0.21 |  |  | No diff |
| Gly m TI | 14.01±0.21 |  |  | No diff |
| Jug n 1 | 15.12±0.52 | *Juglans nigra* | 13.72±0.12 | Greater |
| Jug n 2 | 15.79±0.97 |  |  | No diff |
| Jug r 1 | 15.31±0.51 | *Juglans regia* | 13.95±0.16 | Greater |
| Jug r 2 | 14.68±0.20 |  |  | Greater |
| Lyc e 2 | 13.89±0.20 | *Lycopersicon esculentum* | 13.75±0.01 | No diff |
| Lyc e 3 | 12.11±0.24 |  |  | Smaller |
| Lyc e NP24 | 13.12±0.23 |  |  | No diff |
| Mal d 2 | 14.18±0.21 | *Malus domestica* | 13.55±0.02 | Greater |
| Mal d 3 | 12.40±0.22 |  |  | Smaller |
| Met e 1 | 17.98±0.67 | *Metapenaeus ensis* | 13.53±0.11 | Greater |
| Mim n 1 | 27.17±2.82 | *Mimachlamys nobilis* | 16.49±1.05 | Greater |
| Ory s TAI | 12.26±0.10 | *Oryza sativa* | 13.49±0.01 | Smaller |
| Sal s 1 | 12.65±0.15 | *Salmo salar* | 12.15±0.14 | No diff |
| Ses i 1 | 15.20±0.39 | *Sesamum indicum* | 13.64±0.06 | Greater |
| Ses i 2 | 15.75±0.81 |  |  | No diff |
| Ses i 3 | 22.67±2.55 |  |  | Greater |
| Ses i 4 | 12.03±0.21 |  |  | Smaller |
| Ses i 5 | 12.16±0.12 |  |  | Smaller |
| Sin a 1 | 15.52±0.41 | *Sinapis alba* | 12.84±0.08 | Greater |
| Sola t 2 | 12.48±0.23 | *Solanum tuberosum* | 11.86±0.09 | No diff |
| Sola t 3 | 12.37±0.22 |  |  | No diff |
| Sola t 4 | 12.80±0.22 |  |  | Greater |
| Tri a 18 | 13.55±0.28 | *Triticum aestivum* | 13.50±0.01 | No diff |
| Tri a gliadin | 15.50±0.16 |  |  | Greater |
| Tri a glutenin | 15.44±0.19 |  |  | Greater |
| Zea m 14 | 12.21±0.22 | *Zea mays* | 13.48±0.01 | Smaller |
