# Supplementary material for: Computational analysis of the relationship between allergenicity and digestibility of allergenic proteins in simulated gastric fluid
Source: BMC Bioinformatics. 2007 Oct 9;8:375. doi: 10.1186/1471-2105-8-375 (PMC2099448; doi:10.1186/1471-2105-8-375)
Supplement: Additional file 2 — The digestibility of NACS food allergens and relevant SOPS. The data provided represent the digestibility of NACS food allergens and relevant SOPS. [file 1471-2105-8-375-S2.doc]

## Table 4 - The digestibility of NACS food allergens and relevant SOPS

Digestibility: the unit is the amino acid residue.

Comparison: comparing the digestibility of NACS food allergens with the digestibility of relevant SOPS by t-test (α= 0.01).

| Allergens | Digestibility | Species | Digestibility | Comparison |
| --- | --- | --- | --- | --- |
| Act c 1 | 13.70±0.22 | *Actinidia chinensis* | 13.44±0.06 | No diff |
| Api g 1 | 12.35±0.21 | *Apium graveolens* | 13.23±0.05 | Smaller |
| Api g 4 | 12.08±0.22 |  |  | Smaller |
| Api g 5 | 11.18±0.27 |  |  | Smaller |
| Ara h 5 | 11.82±0.21 | *Arachis hypogaea* | 13.61±0.04 | Smaller |
| Ara h 8 | 13.60±0.15 |  |  | No diff |
| Bos d 2 | 13.69±0.23 | *Bos domesticus* | 13.98±0.01 | No diff |
| Bos d 3 | 12.55±0.27 |  |  | Smaller |
| Bra n 1 | 14.86±0.26 | *Brassica napus* | 13.62±0.02 | Greater |
| Bra n 2 | 13.44±0.33 |  |  | No diff |
| Bra r 1 | 14.15±0.34 | *Brassica rapa* | 13.62±0.03 | No diff |
| Cap a 2 | 11.97±0.21 | *Capsicum annuum* | 13.68±0.03 | Smaller |
| Car p 1 | 13.63±0.24 | *Carica papaya* | 13.30±0.05 | No diff |
| Cor a 1 | 14.20±0.13 | *Corylus avellana* | 13.94±0.14 | No diff |
| Cor a 2 | 11.89±0.22 |  |  | Smaller |
| Dau c 1 | 12.57±0.10 | *Daucus carota* | 12.24±0.12 | No diff |
| Dau c 4 | 12.24±0.23 |  |  | No diff |
| Gly m 1 | 13.87±0.33 | *Glycine max* | 13.69±0.01 | No diff |
| Gly m 2 | 14.81±0.20 |  |  | Greater |
| Gly m 3 | 12.22±0.22 |  |  | Smaller |
| Gly m 4 | 13.30±0.17 |  |  | No diff |
| Hor v 1 | 12.86±0.09 | *Hordeum vulgare* | 13.42±0.01 | Smaller |
| Hor v 9 | 13.16±0.25 |  |  | No diff |
| Lit c 1 | 11.96±0.23 | *Litchi chinensis* | 13.45±0.15 | Smaller |
| Lyc e 1 | 11.94±0.15 | *Lycopersicon esculentum* | 13.75±0.01 | Smaller |
| Lyc e LAT52 | 17.23±0.77 |  |  | Greater |
| Mal d 1 | 14.38±0.21 | *Malus domestica* | 13.55±0.02 | Greater |
| Mal d 4 | 12.09±0.13 |  |  | Smaller |
| Mus xp 1 | 11.81±0.21 | *Musa x paradisiaca* | 13.23±0.14 | Smaller |
| Ory s 1 | 13.90±0.06 | *Oryza sativa* | 13.49±0.01 | Greater |
| Ory s 33kD | 12.42±0.23 |  |  | Smaller |
| Sola t 1 | 13.34±0.17 | *Solanum tuberosum* | 11.86±0.09 | Greater |
| Tri a 3 | 14.97±0.24 | *Triticum aestivum* | 13.50±0.01 | Greater |
| Tri a profilin | 12.07±0.21 |  |  | Smaller |
| Tri a ps93 | 13.26±0.21 |  |  | No diff |
| Tri a TAI | 12.71±0.10 |  |  | Smaller |
| Zea m 1 | 14.18±0.22 | *Zea mays* | 13.48±0.01 | Greater |
